# Ventilator-associated pneumonia in neurocritically ill patients: insights from the ENIO international prospective observational study

**DOI:** 10.1186/s12931-023-02456-9

**Published:** 2023-05-31

**Authors:** Denise Battaglini, Luca Parodi, Raphael Cinotti, Karim Asehnoune, Fabio Silvio Taccone, Giovanni Orengo, Gianluigi Zona, Antonio Uccelli, Giulio Ferro, Michela Robba, Paolo Pelosi, Chiara Robba

**Affiliations:** 1 IRCCS Ospedale Policlinico San Martino, Genoa, Italy; 2 Department of Informatics, Bioengineering, Robotics and Systems Engineering, DIBRIS, Via Opera Pia 13, 16145 Genoa, Italy; 3 Department of Anaesthesia and Critical Care, CHU Nantes, Nantes Université, Hôtel Dieu, 44000 Nantes, France; 4 Department of Intensive Care, Hôpital Universitaire de Bruxelles (HUB), Université Libre de Bruxelles (ULB), Brussels, Belgium; 5 DINOGMI, University of Genoa, Genoa, Italy; 6 Department of Surgical Sciences and Integrated Diagnostics, University of Genoa, Genoa, Italy

**Keywords:** Ventilator-associated pneumonia, Neurocritical care, Brain injury, Pneumonia, Tracheobronchitis, Outcome

## Abstract

**Background:**

Acute brain injured (ABI) patients are at high risk of developing ventilator-associated pneumonia (VAP). However, incidence, risk factors and effects on outcome of VAP are not completely elucidated in this population. The primary aim of this study was to determine the incidence of VAP in a cohort of ABI patients. The secondary objectives included the identification of risk factors for development of VAP, and the impact of VAP on clinical outcomes. Clinical outcomes were defined as intensive care unit length of stay (ICU-LOS), duration of invasive mechanical ventilation (IMV), and ICU mortality.

**Methods:**

Pre-planned sub-analysis of the Extubation strategies in Neuro-Intensive care unit (ICU) patients and associations with Outcomes (ENIO) international multi-center prospective observational study. Patients with available data on VAP, who received at least 48 h of IMV and ICU-LOS ≥ 72 h were included.

**Results:**

Out of 1512 patients included in the ENIO study, 1285 were eligible for this analysis. The prevalence of VAP was 39.5% (33.7 cases /1000 ventilator-days), with a high heterogeneity across countries and according to the type of brain injury. VAP was significantly more frequent in male patients, in those with smoke habits and when intraparenchymal probe (IP), external ventricular drain (EVD) or hypothermia (p < 0.001) were used. Independent risk factors for VAP occurrence were male gender, the use of IP, hypothermia, and the occurrence of tracheobronchitis during ICU stay. VAP was not an independent risk factor for ICU mortality (Hazard Ratio, HR = 0.71 95%CI 0.43–1.16, p = 0.168), but was independently associated with longer ICU stay (OR = 2.55 95%CI 2.01–3.23, p < 0.001).

**Conclusions:**

VAP is common in ABI patients. Male gender, IP and EVD insertion, tracheobronchitis, and the use of therapeutic hypothermia were significantly associated with VAP occurrence. VAP did not affect mortality but increased ICU-LOS.

**Supplementary Information:**

The online version contains supplementary material available at 10.1186/s12931-023-02456-9.

## Background

Ventilator-associated pneumonia (VAP) is the most common nosocomial infection in the intensive care unit (ICU) [1]. VAP is defined as lower respiratory tract infection occurring at least 48 h after initiation of invasive mechanical ventilation (MV) [2]. Acute brain injury (ABI) with impaired consciousness is a risk factor for respiratory complications and VAP development [3]. Swallowing dysfunction, older age, and sedation have been identified as additional and relevant risk factors for the acquisition of pneumonia [3–5]. Small studies reported an incidence of VAP in the ABI population of around 31%, with a VAP rate of 7–18/1000 ventilator-days [6–10]. Moreover, some studies also suggested that VAP occurrence was associated with increased mortality, ICU-length of stay (LOS), and longer duration of MV [11]. However, results of these studies are heterogeneous, and the incidence and risk factors associated with VAP development, and its effects on outcome are still uncertain in this population [12].

The primary aim of this secondary analysis of the Extubation strategies in Neuro-Intensive care unit patients and associations with Outcomes (ENIO) international multi-center prospective observational study was to determine the incidence and rate of VAP in ABI patients. Secondary aims included the assessment of the risk factors for VAP development, and the impact of VAP on patients’ clinical outcome.

## Methods

### Study design

This is a secondary analysis of the ENIO investigator-initiated prospective, multicenter, international, observational study (NCT03400904) [11, 13]. The ENIO study was endorsed and promoted by the PROtective VEntilation Network, European Society of Intensive Care Medicine, Society of Critical Care Medicine, French Society of Anesthesiology and Critical care (SFAR), and Colegio Mexicano de Medicina Critica. Initial approval was obtained from Groupe Nantais d’Éthique dans le Domaine de la Santé (IRB No. 7/11/2017). Approval for conducting the ENIO study in each participating center was obtained by the local medical ethics committees. Informed consent was collected in accordance with the local regulations of each involved IRB, and was obtained directly from the patient, either before the study or retrospectively in case the patient was unconscious at the time of enrolment. This subanalysis was approved by the ENIO steering committee and was conducted according to the Strengthening the Reporting of Observational Studies in Epidemiology (STROBE) reporting guidelines [24] (Additional file 1: Item S1), and in full conformity with the principles of the Declaration of Helsinki, and the Medical Research Involving Human Subjects Act (WMO) [14]. Data management, monitoring and reporting of the study were performed in accordance with the ICH-GCP Guidelines [15].

### Study population

#### Inclusion and exclusion criteria

The inclusion and exclusion criteria of the ENIO study have been previously described in detail [11, 13]. In particular, the ENIO study enrolled neurocritical care patients with traumatic brain injury (TBI), subarachnoid aneurysmal hemorrhage (aSAH), intracranial hemorrhage (ICH), ischemic stroke (IS), and central nervous system (CNS) infection (brain abscess, empyema, meningitis, encephalitis), or brain tumor, aged 18 years/ old who made an attempt of extubation and required invasive mechanical ventilation for at least 24 h, with a baseline Glasgow Coma Score (GCS) of 12 at ICU admission [11, 13]. For this secondary analysis, we additionally selected patients with available data on VAP from the ENIO cohort.

Patients were excluded from the ENIO study if they were under 18 years old, pregnant, had a spinal cord injury above T4, had been resuscitated following a cardiac arrest, had Guillain–Barré syndrome, died prior to extubation, withdrew life-sustaining treatment (WLST) within the first 24 h of ICU admission, undergone end-of-life extubation, had major respiratory co-morbidities (defined as chronic oxygen use at home, chronic obstructive). Patients who had tracheostomies prior to being admitted to the ICU were also excluded. Additionally, for this secondary analysis, we excluded all patients who lasted less than 48 h of invasive mechanical ventilation and ICU stay ≤ 72 h.

### Definitions

VAP was diagnosed according to the American Thoracic Society (ATS) criteria published in 2005 [16]. Diagnosis of VAP was suspected when patient showed a new or progressive radiographic infiltrate, along with clinical findings suggesting infection like new onset of fever, purulent sputum, leukocytosis, and decline in oxygenation.

Tracheobronchitis was defined by presence of fever, leukocytosis, purulent sputum, and a positive culture of a sputum or tracheal aspirate are present without a new lung infiltrate [16].

VAP and tracheobronchitis after spontaneous breathing trial (SBT) were defined as the abovementioned conditions which were diagnosed after an attempt of spontaneous breathing with the aim of extubate the patient. Attempt of extubation was defined as an extubation trial and/or tracheostomy.

### Data collection

Data of the ENIO main study were collected from the 26 of June 2018 to 15 of November 2020. For this secondary analysis, the following data from the ENIO dataset were selected: demographic and baseline (age, gender, height, weight, BMI = body mass index, previous comorbidities [COPD = chronic obstructive pulmonary disease, cardiovascular comorbidities defined as NYHA = New York Health Association ≥ 2, arterial hypertension, active smoking, diabetes mellitus, history of malignancy]); type (TBI, aSAH, IS, ICH, CNS infection, and brain tumor) and severity (baseline lower GCS) of brain injury; neurosurgical and neurocritical care management (barbiturate coma, therapeutic hypothermia, external ventricular drainage, decompressive craniectomy, and location of cerebral injury [posterior fossa]); airway and ventilatory management data (tracheostomy, gag reflex, cough, spontaneous breathing trial, extubation, reintubation), type of ventilation and ventilatory setting [V_T_ = tidal volume (mL), P_PLAT_ = plateau pressure (cmH_2_O), RR = respiratory rate (breaths/min), PEEP = positive end-expiratory pressure (cmH_2_O)] at day 1, 3, and 7 of ICU admission, gas exchange [pHa, arterial partial pressure of oxygen = PaO_2_ (mmHg), fraction of inspired oxygen = FiO_2_, arterial partial pressure of carbon dioxide = PaCO_2_ (mmHg)] at day 1, 3, and 7 of ICU admission; in-ICU events (tracheobronchitis, ARDS = acute respiratory distress syndrome, WLST = withdrawn life sustaining therapies); outcome measures (need and duration of invasive mechanical ventilation = IMV, ICU-LOS, in-ICU mortality, in-hospital mortality, need of non-invasive mechanical ventilation = NIMV and duration). VAP diagnosis (yes, no) during ICU-stay.

### Study objectives

The primary objective of this sub-study of the ENIO cohort was to assess the prevalence and rate (cases/1000 ventilator days at risk) of VAP among mechanically ventilated adult patients with ABI. The secondary objectives included the identification of risk factors for development of VAP, and the impact of VAP on clinical outcomes. Clinical outcomes were defined as ICU-LOS, duration of IMV, need for non-invasive mechanical ventilation, and ICU-mortality.

### Statistical analysis

Data were expressed as means (standard deviation = SD), medians [interquartile range = IQR] and proportions when appropriate. Continuous variables were compared by using the Student’s *t*-test or Mann–Whitney U-tests, while categorical variables were analyzed with the Chi-squared test. Shapiro–Wilk test was used to assess the normal distribution of continuous variables.

Logistic regression was performed to assess the risk factors associated with VAP. All the potential risk factors were transformed in dichotomic variables and entered in the univariate analysis, including age, gender, BMI, baseline comorbidities, type of brain injury, severity of brain injury, invasiveness of treatment, country, respiratory complications, and IMV days. The goodness-of-fit evaluation of each significant logistic regression model was performed. Significant variables to univariate logistic regression were entered in the multivariate model, with regression coefficient and odds ratio (OR) with the 95% confidence interval (CI) as the main outputs.

Survival probability has been estimated using the Cox proportional-hazards model. The hazard ratio (HR) was used to assess the likelihood of the event occurring while controlling for other co-predictors (co-variables/co-factors) added to the model. We considered the following predictors in the univariable model: VAP, age, lowest GCS, anisocoria, need for neurosurgery, ICP probe, EVD probe, pulmonary comorbidity, and cardiovascular comorbidity. The significant variables at univariate were entered in the multivariate Cox regression model (VAP, age, ICP probe, cardiovascular comorbidity). The Kaplan–Meier task was used for comparing the survival curves between VAP and noVAP stratified by co-variables/ co-factors.

Statistical significance was considered for a p < 0.05. Statistical analysis was performed with R software.

## Results

**Fig. 1:**
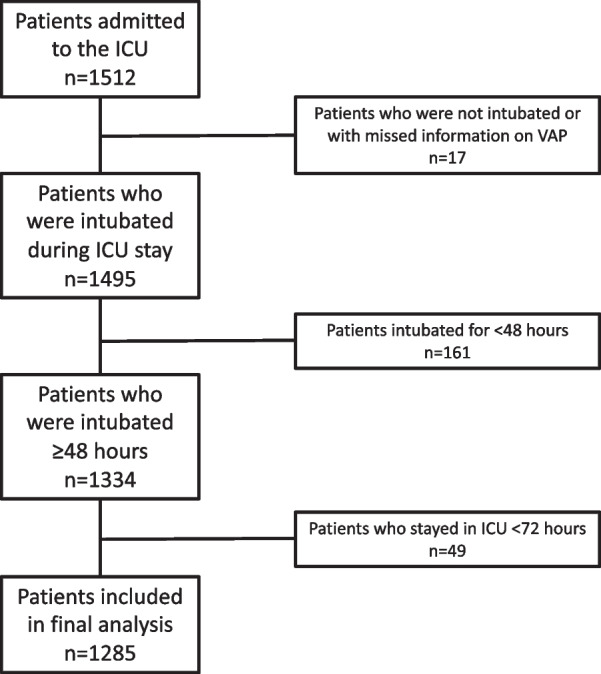
Flow-chart of inclusion

**Fig. 2:**
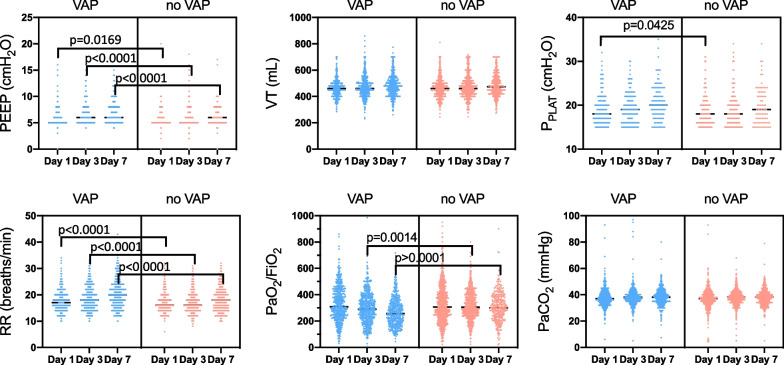
Characteristics of mechanical ventilation and gas exchange at day 1, 3, and 7 of ICU admission. At day 1, 3, and 7 of ICU admission, PEEP was higher in VAP than no VAP [day 1—VAP = 5 (5–6) vs. no VAP = 5 (5–6) cmH_2_O, p = 0.0169; day 3—VAP = 6 (5–7) vs. no VAP = 5 (5–6) cmH_2_O, p < 0.0001; day 7—VAP = 6 (5–8) vs. no VAP = 6 (5–7) cmH_2_O, p < 0.0001]; RR was higher in VAP than no VAP [day 1—VAP = 17 (15–20) vs. no VAP = 16 (14–18) rpm, p < 0.0001; day 3 VAP = 18 (15–22) vs. no VAP = 16 (14–19) rpm, p < 0.0001; day 7—VAP = 20 (16–24) vs. no VAP = 18 (15–21) rpm p < 0.0001], P_PLAT_ was higher in VAP than no VAP [day 1—VAP = 18 (17–21) vs. no VAP = 18 (16–20) cmH_2_O, p = 0.0425 (all flimsy values below 15 cmH_2_O have not been included in the calculation)]; and PaO_2_/FiO_2_ was lower in VAP than no VAP [day 3—VAP = 290 (220–376.2) vs. no VAP = 305 (246.7–396), p = 0.0014; day 7—VAP = 256 (187.1–333.8) vs. no VAP = 300 (233.3–383.3), p < 0.0001]. V_T_ and PaCO_2_ did not significantly change between groups over time. *ICU* intensive care unit, *PEEP* positive end-expiratory pressure, *VAP* ventilator-associated pneumonia, *RR* respiratory rate, *VT* tidal volume, *PaCO*_*2*_ arterial partial pressure of carbon dioxide

A total of 1512 patients were included in the initial cohort; overall, 227 patients had missing data on VAP, were ventilated for ≤ 48 h or had an ICU stay ≤ 72 h and were excluded from the analysis. The final cohort included 1285 ABI patients (Fig. 1)**.** Characteristics of the study population are reported in Table 1, and data on mechanical ventilation and gas exchange are reported in Fig. 2 and Additional file 1: Item S2.

**Table 1:** Baseline characteristics, demographics and ICU outcomes of the overall population and according to the occurrence of VAP

	Overall n = 1285	VAP n = 550	noVAP n = 735	p-value
Demographics				
Age, years, median [IQR]	54 [37–66]	54 [36–65]	55 [37–67]	0.386
Gender, n (%)				
Male	838 (65.2)	382 (45.6)	456 (54.4)	**0.006**
Female	447 (34.8)	168 (37.6)	279 (62.4)	
BMI, kg/m^2^, median [IQR]	25 [23–29]	26 [23–29]	25 [22–28]	0.071
Baseline comorbidities				
COPD	42 (3.3)	22 (52.4)	20 (47.6)	0.200
NYHA ≥ 2	38 (3)	15 (39.5)	23 (60.5)	0.678
Hypertension	386 (30)	159 (41.2)	227 (58.8)	0.457
Active smoking	285 (22.2)	140 (49.1)	145 (50.9)	**0.013**
Diabetes mellitus	156 (12.1)	68 (43.6)	88 (56.4)	0.823
History of malignancy	60 (4.7)	25 (41.7)	35 (58.3)	0.861
Cause of ICU admission				
TBI, n (%)	618 (48.1)	272 (44)	346 (56)	0.398
ICH, n (%)	438 (34.1)	179 (40.9)	259 (59.1)	0.314
SAH, n (%)	235 (18.3)	114 (48.5)	121 (51.5)	0.051
Ischemic Stroke, n (%)	115 (8.9)	39 (33.9)	76 (66.1)	**0.043**
CNS infection, n (%)	62 (4.8)	13 (21)	49 (79)	**< 0.001**
Brain Tumor, n (%)	59 (4.6)	18 (30.5)	41 (69.5)	0.051
Neurologic characteristics				
GCS total, median [IQR]	7 [5–9]	7 [5–8]	7 [5–9]	0.309
Anisocoria, n (%)	355 (27.6)	161 (45.4)	194 (54.6)	0.229
Intraparenchymal probe, n (%)	590 (45.9)	318 (53.9)	272 (46.1)	**< 0.001**
EVD, n (%)	398 (31)	201 (50.5)	197 (49.5)	**< 0.001**
Posterior fossa injury, n (%)	79 (6.1)	38 (48.1)	41 (51.9)	0.322
Therapeutic hypothermia, n (%)	60 (4.7)	45 (75)	15 (25)	**< 0.001**
Barbiturate coma, n (%)	78 (6.1)	38 (48.7)	40 (51.3)	0.276
Intra-cranial neurosurgery, n (%)	522 (40.6)	229 (43.9)	293 (56.1)	0.535
Decompressive craniectomy, n (%)	244 (19)	115 (47.1)	129 (52.9)	0.129
ICU outcome				
VAP after SBT, n (%)	176 (13.7)	164 (93.2)	12 (6.8)	**< 0.001**
Tracheobronchitis after SBT, n (%)	128 (10)	78 (60.9)	50 (39.1)	**< 0.001**
ARDS, n (%)	128 (10)	113 (88.3)	15 (11.7)	**< 0.001**
Tracheostomy required, n (%)	347 (27)	179 (51.6)	168 (48.4)	**< 0.001**
IMV duration, days, median [IQR]	9 [5–17]	14 [8–22]	6 [4–11]	**< 0.001**
Non-IMV, n (%)	161 (12.5)	96 (59.6)	65 (40.4)	**< 0.001**
HFNO, n (%)	229 (17.8)	124 (54.1)	105 (45.9)	**< 0.001**
ICU LOS, days, median [IQR]	15 [9–25]	22 [14–32]	11 [7–18]	**< 0.001**
WLST, n (%)	78 (6.1)	35 (44.9)	43 (55.1)	0.712
In-ICU mortality, n (%)	71 (5.5)	37 (52.1)	34 (47.9)	0.104
In-hospital mortality, n (%)	137 (10.7)	66 (48.2)	71 (51.8)	0.175

Bold stands for statistical significative values

Data are presented as median [interquartile range = IQR] and n = number (percentages = %). *BMI* body mass index, *VAP* ventilator-associated pneumonia, *COPD* chronic obstructive pulmonary disease, *NYHA* New York Health Association, *TBI* traumatic brain injury, *ICH* intracranial hemorrhage, *SAH* subarachnoid hemorrhage, *CNS* central nervous system, *GCS* Glasgow coma scale, *EVD* external ventricular device, *SBT* spontaneous breathing trial, *ARDS* acute respiratory distress syndrome, *IMV* invasive mechanical ventilation, *HFNO* high flow nasal oxygen, *ICU* intensive care unit, *LOS* length of stay, *WLST* withdrawn life sustains therapies

### Prevalence and rate of VAP

A total of 550 (39.5%) patients were diagnosed with VAP during their ICU stay; the rate of VAP was 33.7/1000 ventilator-days. Patients with VAP were more often males; active smokers, were more frequently monitored with intraparenchymal probe or EVD; and more often treated with hypothermia than others (Table 1).

**Fig. 3:**
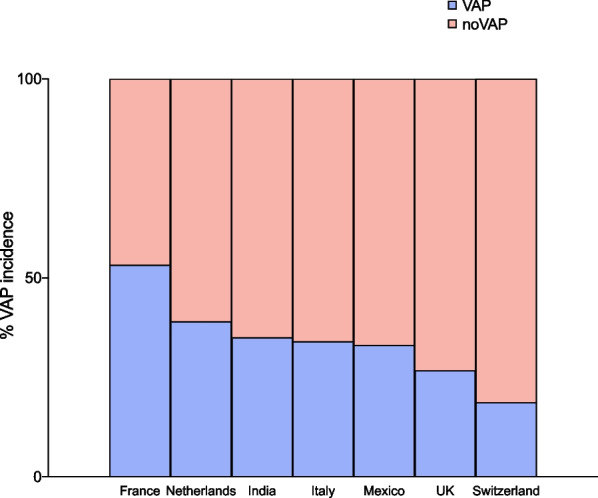
VAP incidence per country. This figure depicts the incidence of VAP according to the countries included in the ENIO study. Countries with ≥ 50 patients were included

At day 1, 3, and 7 after ICU admission, PEEP, and RR were significantly higher while PaO_2_/FiO_2_ was significantly lower in VAP than no-VAP group; V_T_ and PaCO_2_ did not significantly change between groups over time; P_PLAT_ was higher at day 1 in VAP than noVAP group (Additional file 1: Item S2). The prevalence and rate of VAP was 49.5% (n = 272)—16.6/1000 ventilator-days in TBI, 32.5% (n = 179)—11.0/1000 ventilator-days in ICH, 20.7% (n = 114)—7.0/1000 ventilator-days in aSAH, 3% (n = 39)—7.1/1000 ventilator-days in IS, 3.3% (n = 18)—1.1/1000 ventilator-days in brain tumor, and 2.4% (n = 13)—0.8/1000 ventilator-days in CNS infection, respectively. The prevalence of VAP was higher in France (53.3%), followed by Netherlands (39%), India (35%), Italy (34%), Mexico (33.1%), UK (26.7%), and Switzerland (18.8%), Fig. 3.

### Factors associated with VAP

At univariate analysis, factors associated with VAP development are reported in Additional file 1: Item S3. In the multivariable model, independent risk factors for VAP occurrence were male gender, the use of intraparenchymal probe, (OR = 2.05 95%CI 1.62–2.61, p < 0.001), EVD, (OR = 1.45 95%CI 1.12–1.88, p = 0.004), hypothermia (OR = 3.06 95%CI 1.65–5.68, p < 0.001), and occurrence of tracheobronchitis after spontaneous breathing trial (OR = 2.26 95%CI 1.53–3.32, p < 0.001). CNS infections was associated with a significant reduction of the risk of VAP development (Table 2).

**Table 2:** Multivariable analysis: risk factors for VAP development

	Estimate	Std. Error	z value	Pr( >|z|)	Std. Coeff	OR	95%CI
(Intercept)	− 0.7042	0.09886	− 7.123	< 0.0001	− 0.7042	0.4945	[0.41–0.60]
Female gender	− 0.43069	0.12768	− 3.373	< 0.0001	− 0.4307	0.6501	[0.51–0.83]
CNS infection	− 0.83772	0.32676	− 2.564	= 0.0104	− 0.8377	0.4327	[0.23–0.82]
Intraparenchymal probe	0.72024	0.12139	5.933	< 0.0001	0.7202	2.0549	[1.62–2.61]
External ventricular device	0.373	0.13086	2.85	= 0.0044	0.3730	1.4521	[1.12–1.88]
Therapeutic hypothermia	1.11978	0.31451	3.56	< 0.0001	1.1198	3.0642	[1.65–5.68]
Tracheobronchitis at spontaneous breathing trial	0.81327	0.19805	4.106	< 0.0001	0.8133	2.2553	[1.53–3.32]

*CNS* central nervous system, *OR* odds ratio, *CI* confidence interval, *Pr* probability, *Std. Error* standard error, *Std. Coefficient* standard coefficient

### Effect of VAP on outcomes

Compared to patients without VAP, those with VAP had longer ICU length of stay (median 22 [IQR = 14–32] vs. 11 [IQR = 7–18] days, p < 0.001), duration of invasive mechanical ventilation (median 14 [IQR = 8–22] vs. 6 [IQR = 4–11] days, p < 0.001), required more frequently tracheostomy (179, 32.5% vs. 168, 22.9%, p < 0.001), non-invasive mechanical ventilation (n = 96, 17.5 vs. 65, 8.8%, p < 0.001), high-flow nasal oxygen therapy (n = 124, 22.5% vs. 105, 14.3%, p < 0.001) and had a higher incidence of ARDS (113, 20.5% vs. 15, 2%, p < 0.001), and tracheobronchitis (n = 78, 14.2% vs. 50, 6.8%, p < 0.001).

One patient missed ICU mortality outcome. Mortality in ICU and in-hospital did not differ between patients who experienced or not VAP (ICU: 37 deaths, 52.1% vs. 34 deaths, 49.7%; p = 0.104—Hospital: 66 deaths, 48.2% vs. 71 deaths, 51.8%; p = 0.175].

At univariate Cox regression model, high age and cardiovascular comorbidity resulted associated with increased ICU mortality (Additional file 1: Item S4). No association was found for lower GCS, anisocoria, need for neurosurgery, EVD probe, and pulmonary comorbidity) and ICU mortality.

**Fig. 4:**
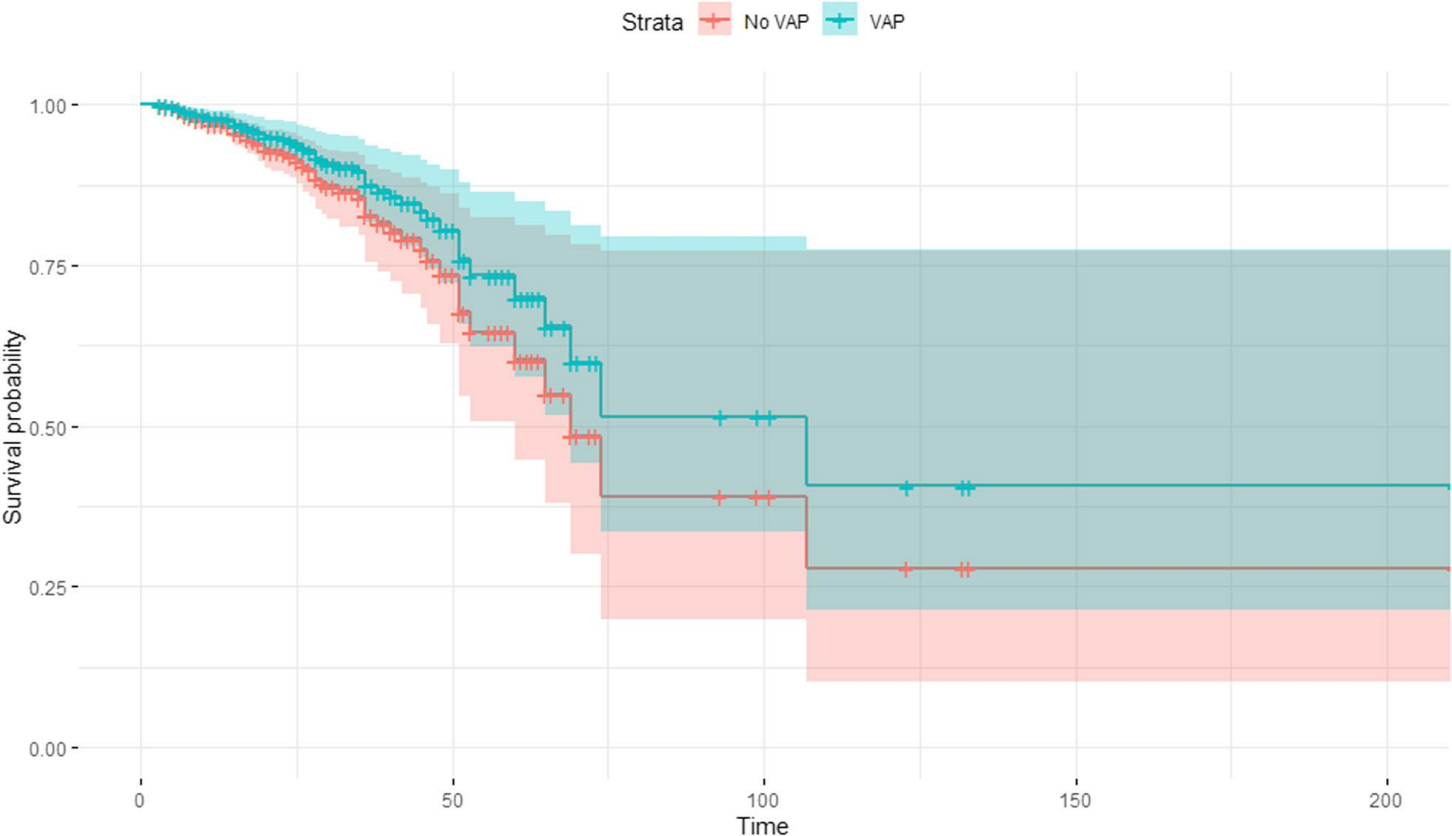
Survival estimates in ICU of patients with and without VAP. Kaplan Meier survival estimates of patients with and without VAP in ICU. Kaplan Meier was adjusted for relative hazard of covariates used in the Cox regression model. HR = hazard ratio

At multivariate Cox regression model, the presence of VAP was not independently associated with increased ICU mortality (HR = 0.71 95%CI 0.43–1.16, p = 0.168), whereas higher age and presence of cardiovascular comorbidity were independently associated with increased ICU mortality (age: HR = 1.03 95%CI 1.01–1.05, p < 0.0001; cardiovascular: HR = 2.75 95%CI 1.23–6.14, p = 0.013). The presence of ICP probe was independently associated with reduced ICU mortality (HR = 0.31 95%CI 0.17–0.55, p < 0.0001) (Additional file 1: Item S5). Kaplan Meier curve of cumulative survival in VAP versus noVAP groups is shown in Fig. 4.

The occurrence of VAP (OR = 2.55 95%CI 2.01–3.23, p < 0.001) and the duration of invasive mechanical ventilation (OR = 8.1 95%CI 6.72–9.75, p < 0.001) were independent risk factors for longer ICU-stay (Additional file 1: Item S6).

## Discussion

The main findings of this study, in a cohort of 1285 brain injured patients, are: (1) VAP prevalence and rate is high in ABI patients, especially in TBI. Specifically, the prevalence and rate of VAP was 49.5%–16.6/1000 ventilator-days in TBI, 32.5%–11.0/1000 ventilator-days in ICH, 20.7%–7.0/1000 ventilator-days in aSAH, and 3%–7.1/1000 ventilator-days in IS; (2) independent risk factors for VAP development in brain injured patients include male gender, intraparenchymal probe, EVD, therapeutic hypothermia, and occurrence of tracheobronchitis. CNS infection compared to other ABIs was protective; (3) VAP was not associated with increased ICU mortality, but longer ICU stay and duration of mechanical ventilation.

To the best of our knowledge, this study represents the largest observational study investigating VAP in an international prospective cohort of neurocritically ill patients. VAP is a very common healthcare-associated infection in patients who are critically ill and who require invasive mechanical ventilation.

### Prevalence, rate, and characteristics of patients with VAP

The literature reports an incidence of VAP in mechanically ventilated patients ranging from 10–40%, with a rate between 7.3 and 20.4/1000 ventilator-days [2, 6, 17, 18].

In our cohort, both the incidence and rate of VAP were higher than in previous findings (39.5% and 33.7/1000 ventilator-days respectively) [19], especially in the TBI subgroup (49.5% vs. 15–36%). These differences can be explained by several reasons. We used the currently accepted definition of VAP developed in late 2017 [2], the same year in which the ENIO study started recruiting. Therefore, the adherence to the new VAP definition could have been different across countries and compared with previous studies.

Second, neurological injuries in our cohort were heterogeneous and mainly composed by TBI, ICH, and SAH patients thus making difficult to compare our results with other studies [6, 17, 18]. Moreover, the ENIO cohort had a higher age and more comorbidities than previous cohorts of younger patients with TBI following road traffic accident, thus predisposing to longer duration of mechanical ventilation, which is clear risk factor for VAP, and complications [12, 20, 21].

However, in SAH, we found a 20.7% VAP incidence, which is lower in comparison with previous findings (up to 49%) [22–24]. This may be due to the fact that current guidelines suggest that patients with SAH are managed trying to keep them awake as much as possible to early detected a possible clinical deterioration (i.e., vasospasm) through assessment of sequential neurological examinations during ICU stay. This may impact on earlier extubation and reduced days of mechanical ventilation, thus decreasing the risk of developing VAP [25–27]. In our cohort, we observed that only 3% of patients with ischemic stroke developed VAP, which is lower than reported in previous studies (4–57%) [28]. This different incidence can be explained by the fact that previous studies included cohorts of non-critically ill patients with stroke with both healthcare associated pneumonia and VAP [29]. We also found a 3.3% prevalence of VAP in brain tumor, and 2.4% in CNS infection, but no previous data have been reported the literature in these sub-groups of neurocritically ill patients.

Some literature reports lower VAP rates than our study probably because of strict adherence to an oral care “bundle”, a selection bias in relatively healthy patients who were intubated for only a few days post-operatively, and mortality from neurologic disease prior to the development of VAP [23]. Another possible explanation can be the neurological status upon admission, that in our study was more severe than in previous studies, meaning that our patients required more treatment for neurological injury (patients with VAP more frequently needed intraparenchymal probe, EVD, and therapeutic hypothermia), which may result in higher therapy intensity level and therefore longer days of treatment and mechanical ventilation [6, 12]. Finally, our results suggest also that VAP is more frequent in males and active smokers, that is in line with previous investigations [30] which underline the importance of pre-existing comorbidities and susceptibility to lung damage. VAP has an important impact on systemic oxygenation, thus worsening mechanical ventilator parameters but no effects on carbon dioxide. PaO_2_/FiO_2_, levels between day 1 to 7 suggested no significative impact on brain physiology and neurological outcome [31, 32]. Also, intubation strategies may have impacted, maybe some patients tolerated GCS 7–8 without intubation possibly being not at risk of VAP.

### Factors associated with VAP

Risk factors for VAP occurrence in our cohort were male gender, the use of ICP monitoring, EVD, therapeutic hypothermia, and the occurrence of tracheobronchitis after spontaneous breathing trial. On the other hand, CNS infection was protective for VAP development. The prevalence of intraparenchymal probe and EVD was higher in VAP group and independently increased the risk of VAP. Pelosi et al. reported that VAP was more frequent in brain damaged patients than general critically ill population, suggesting that neurological severity and the need of invasive monitoring and aggressive treatment may have an influence [33]. Indeed, patients with VAP were more clinically severe than no VAP, undergoing more tracheostomy, longer duration of mechanical ventilation, ICU-stay, ARDS, and tracheobronchitis [12, 21], although the neurological status was similar between the two groups. In our cohort, therapeutic hypothermia was independently associated with VAP. In the Eurotherm trial, early hypothermia plus standard of care for the control of intracranial hypertension was associated with worst outcome than standard of care alone, suggesting that VAP risk increases in more severe patients who need more aggressive treatments, especially when used early and not as tier three therapy [34]. According to the literature, tracheobronchitis after spontaneous breathing trial was independent risk factor VAP [35–37]. Female gender was less susceptible to VAP development. Previous studies showed that, despite females usually manifest less VAP than males, severe VAP is an independent predictor of mortality in females, especially when diagnosed within 7 days from admission [38].

### VAP effect on patients’ outcomes

Our estimated attributable mortality for VAP was slightly lower than that reported in generally critically ill patients, but ENIO cohort includes patients who were successfully extubated [39]. In the VAP group, we found a mortality of 52% in ICU and 48% in hospital, which were higher than previous works which reported an ICU mortality around 25–35% [20, 23]. Despite that, VAP was not independent risk factor for mortality in ICU (HR = 0.71 95%CI 0.43–1.16, p = 0.168). Similar to our findings, other studies in neurocritically ill patients reported no association between VAP and mortality [23, 24]. This is because VAP may be only a transient disease early detected in ICU and treated following an appropriate antibiotic stewardship [40], which complicates the acute phase of neurological illness, unlikely mortality. Moreover, especially in the European setting, the advances in antibiotic stewardship have improved the course of healthcare infections, thus impacting on outcome [41]. Interventions like re-education of neuro-ICU personnel, and reduction of transports for brain imaging can help in reducing the rate of infection [42]. VAP was independent risk factor for longer ICU length of stay and duration of invasive mechanical ventilation. This is an important point, as it suggests that VAP can have an important impact on costs and healthcare resources utilization [12, 23].

### Limitations

This study has several limitations that must be addressed. Firstly, the main limitation of our study is the observational design, which cannot provide information about causality but only associations. Second, this is a secondary analysis of the main study whose primary outcome was to describe current management of weaning from invasive ventilation, focusing on decisions on timing of tracheal extubation and tracheostomy, where VAP represents a secondary outcome. Third, the given the unavailability of timing of VAP diagnosis, this limited a possible distinction in early vs. late VAP, and probably led to an overestimation. Fourth, no data on antibiotics use and type, as well as pathogens and diagnostic tests and other ICU therapies that may have affected VAP development were collected in the main ENIO study. Fifth, scores of severity such as APACHE or SOFA at ICU admission were not available, thus GCS, pupillary reactivity, and invasiveness of treatment (i.e., EVD, ICP monitoring, barbiturates, therapeutic hypothermia, decompressive craniectomy, and need for neurosurgery) were considered as indicators of severity.

## Conclusions

VAP is common in neurocritically ill patients but highly variable across countries and type of brain injury. Male gender, intracranial probe, EVD, tracheobronchitis, and therapeutic hypothermia impact on VAP. VAP not clearly influence mortality but ICU-LOS and duration of mechanical ventilation. Further studies accounting for antibiotic use and isolated pathogens in large cohorts of neurocritically ill patients should be guaranteed.

## Supplementary Information

**Additional file 1: Item S1.** Strengthening the Reporting of Observational Studies in Epidemiologyreporting guidelines.** Item S2.** Characteristics of ventilation at day 1, 3, and 7 of ICU admission.** Item S3.** Univariate analysis - risk factors for VAP development.** Item S4.** Logistic Cox univariate regression model for hazard of ICU- mortality.** Item S5.** Logistic Cox multivariate regression model for hazard of ICU- mortality.** Item S6.** Multivariate model risk factors for ICU- length of stay.

## Data Availability

Data will be available to corresponding author under reasonable request.

## Abbreviations

ARDS: Acute respiratory distress syndrome
BMI: Body mass index
CI: Confidence interval
CNS: Central nervous system
COPD: Chronic obstructive pulmonary disease
EVD: External ventricular drain
GCS: Glasgow coma scale
ICH: Intracranial hemorrhage
ICU: Intensive care unit
IMV: Invasive mechanical ventilation
IP: Intraparenchymal probe
IS: Ischemic stroke
LOS: Lenght of stay
NIMV: Non-invasive mechanical ventilation
NYHA: New York Health Association
OR: Odds ratio
PaCO2: Arterial partial pressure of carbon dioxide
PaO2: Arterial partial pressure of oxygen
PEEP: Positive end-expiratory pressure
Pplat: Plateau pressure
RR: Respiratory rate
SAH: Subarachnoid hemorrhage
SD: Standard deviation
TBI: Traumatic brain injury
VAP: Ventilator-associated pneumonia
VT: Tidal volume
WLST: Withdrawn life sustaining therapies
WMO: Medical Research Involving Human Subjects Act
