# Supplementary material for: Ventilator-associated pneumonia in neurocritically ill patients: insights from the ENIO international prospective observational study
Source: Respir Res. 2023 May 31;24:146. doi: 10.1186/s12931-023-02456-9 (PMC10234099; doi:10.1186/s12931-023-02456-9)
Supplement: Supplementary file 1 — Additional file 1: Item S1. Strengthening the Reporting of Observational Studies in Epidemiologyreporting guidelines. Item S2. Characteristics of ventilation at day 1, 3, and 7 of ICU admission. Item S3. Univariate analysis - risk factors for VAP development. Item S4. Logistic Cox univariate regression model for hazard of ICU- mortality. Item S5. Logistic Cox multivariate regression model for hazard of ICU- mortality. Item S6. Multivariate model risk factors for ICU- length of stay. [file 12931_2023_2456_MOESM1_ESM.docx]

Additional File

Ventilator-associated pneumonia in neurocritically ill patients. Insights from the ENIO international prospective observational study

Table of content:

Item S1 - Strengthening the Reporting of Observational Studies in Epidemiology (STROBE) reporting guidelines. [pages 2-3]

Item S2 - Characteristics of ventilation at day 1, 3, and 7 of ICU admission. [page 4]

Item S3 - Univariate analysis - risk factors for VAP development. [page 5]

Item S4 – Logistic Cox univariate regression model for hazard of ICU- mortality. [page 6]

Item S5 - Logistic Cox multivariate regression model for hazard of ICU- mortality [page 6]

Item S6 - Multivariate model risk factors for ICU- length of stay. [page 7]

Item S1 - Strengthening the Reporting of Observational Studies in Epidemiology (STROBE) reporting guidelines.

|  | Item No | Recommendation |
| --- | --- | --- |
| Title and abstract | 1 | (*a*) Indicate the study’s design with a commonly used term in the title or the abstract |
|  |  | (*b*) Provide in the abstract an informative and balanced summary of what was done and what was found |
| Introduction | | |
| Background/rationale | 2 | Explain the scientific background and rationale for the investigation being reported |
| Objectives | 3 | State specific objectives, including any prespecified hypotheses |
| Methods | | |
| Study design | 4 | Present key elements of study design early in the paper |
| Setting | 5 | Describe the setting, locations, and relevant dates, including periods of recruitment, exposure, follow-up, and data collection |
| Participants | 6 | (*a*) *Cohort study*—Give the eligibility criteria, and the sources and methods of selection of participants. Describe methods of follow-up  *Case-control study*—Give the eligibility criteria, and the sources and methods of case ascertainment and control selection. Give the rationale for the choice of cases and controls  *Cross-sectional study*—Give the eligibility criteria, and the sources and methods of selection of participants |
|  |  | (*b*) *Cohort study*—For matched studies, give matching criteria and number of exposed and unexposed  *Case-control study*—For matched studies, give matching criteria and the number of controls per case |
| Variables | 7 | Clearly define all outcomes, exposures, predictors, potential confounders, and effect modifiers. Give diagnostic criteria, if applicable |
| Data sources/ measurement | 8* | For each variable of interest, give sources of data and details of methods of assessment (measurement). Describe comparability of assessment methods if there is more than one group |
| Bias | 9 | Describe any efforts to address potential sources of bias |
| Study size | 10 | Explain how the study size was arrived at |
| Quantitative variables | 11 | Explain how quantitative variables were handled in the analyses. If applicable, describe which groupings were chosen and why |
| Statistical methods | 12 | (*a*) Describe all statistical methods, including those used to control for confounding |
|  |  | (*b*) Describe any methods used to examine subgroups and interactions |
|  |  | (*c*) Explain how missing data were addressed |
|  |  | (*d*) *Cohort study*—If applicable, explain how loss to follow-up was addressed  *Case-control study*—If applicable, explain how matching of cases and controls was addressed  *Cross-sectional study*—If applicable, describe analytical methods taking account of sampling strategy |
|  |  | (*e*) Describe any sensitivity analyses |

| Results | | |
| --- | --- | --- |
| Participants | 13* | (a) Report numbers of individuals at each stage of study—eg numbers potentially eligible, examined for eligibility, confirmed eligible, included in the study, completing follow-up, and analysed |
|  |  | (b) Give reasons for non-participation at each stage |
|  |  | (c) Consider use of a flow diagram |
| Descriptive data | 14* | (a) Give characteristics of study participants (eg demographic, clinical, social) and information on exposures and potential confounders |
|  |  | (b) Indicate number of participants with missing data for each variable of interest |
|  |  | (c) *Cohort study*—Summarise follow-up time (eg, average and total amount) |
| Outcome data | 15* | *Cohort study*—Report numbers of outcome events or summary measures over time |
|  |  | *Case-control study—*Report numbers in each exposure category, or summary measures of exposure |
|  |  | *Cross-sectional study—*Report numbers of outcome events or summary measures |
| Main results | 16 | (*a*) Give unadjusted estimates and, if applicable, confounder-adjusted estimates and their precision (eg, 95% confidence interval). Make clear which confounders were adjusted for and why they were included |
|  |  | (*b*) Report category boundaries when continuous variables were categorized |
|  |  | (*c*) If relevant, consider translating estimates of relative risk into absolute risk for a meaningful time period |
| Other analyses | 17 | Report other analyses done—eg analyses of subgroups and interactions, and sensitivity analyses |
| Discussion | | |
| Key results | 18 | Summarise key results with reference to study objectives |
| Limitations | 19 | Discuss limitations of the study, taking into account sources of potential bias or imprecision. Discuss both direction and magnitude of any potential bias |
| Interpretation | 20 | Give a cautious overall interpretation of results considering objectives, limitations, multiplicity of analyses, results from similar studies, and other relevant evidence |
| Generalisability | 21 | Discuss the generalisability (external validity) of the study results |
| Other information | | |
| Funding | 22 | Give the source of funding and the role of the funders for the present study and, if applicable, for the original study on which the present article is based |

Item S2. Characteristics of ventilation at day 1, 3, and 7 of ICU admission.

|  | VAP | | | No VAP | | |
| --- | --- | --- | --- | --- | --- | --- |
|  | D1 | D3 | D7 | D1 | D3 | D7 |
| Ventilator mode |  |  |  |  |  |  |
| NA, n (%) | 1.27% | 2.18% | 15.09% | 0.68% | 11.29% | 48.71% |
| Pressure assist, n (%) | 7.64% | 23.64% | 26.73% | 12.52% | 23.67% | 20.41% |
| Pressure control, n (%) | 14.91% | 12.55% | 8.00% | 20.41% | 12.93% | 5.99% |
| Spontaneous breathing, n (%) | 1.64% | 5.82% | 13.09% | 1.50% | 11.84% | 10.75% |
| Volume assist, n (%) | 74.55% | 55.82% | 37.09% | 64.90% | 40.27% | 14.15% |
| Ventilator setting |  |  |  |  |  |  |
| V_T_ (mL), median [IQR] | 460 [420 - 500] | 460 [420 - 508.25] | 480 [430 - 525.5] | 460 [420 - 500] | 460 [420 - 517.5] | 474 [420 - 530] |
| PEEP (cmH_2_O), median [IQR] | 5 [5 - 6]* | 6 [5 - 7]* | 6 [5 - 8]* | 5 [5 - 6]* | 5 [5 - 6]* | 6 [5 - 7]* |
| Respiratory rate (breaths/min), median [IQR] | 17 [15 - 20]§ | 18 [15 - 21.75]§ | 20 [16 - 24]§ | 16 [14 - 18]§ | 16 [14 - 19]§ | 18 [15 - 21]§ |
| P_PLAT_ (cmH_2_O), median [IQR] | 18 [17 - 21]° | 19 [17 - 22] | 20 [18 - 23] | 18 [16 - 20]° | 18 [16 - 20] | 18 [16 - 22]° |
| Gas exchange |  |  |  |  |  |  |
| PaO_2_/FiO_2,_ median [IQR] | 308.5 [236.88 - 425] | 290 [220 - 376.19] | 256 [187.5 - 333.33]^ | 306.67 [227.5 - 416] | 305 [246.67 - 396] | 300 [233.33 - 383.1]^ |
| PaCO_2_ (mmHg), median [IQR] | 37 [34 - 41] | 38 [35 - 41] | 38 [35 - 42] | 37 [34 - 41] | 38 [34 - 41] | 38 [34 - 41] |

D=day, NA=not available, VT=tidal volume, PEEP=positive end-expiratory pressure, P_PLAT_=plateau pressure, PaO_2_=arterial partial pressure of oxygen, FiO_2_=fraction of inspired oxygen, PaCO_2_=arterial partial pressure of carbon dioxide.

*significant difference at D1 (p=0.0169), D3 (p<0.0001), and D7 (p<0.0001) between VAP and no VAP.

°significant difference at D3 (p=0.0002) and D7 (p<0.0001) between VAP and no VAP.

§significant difference at D1 (p=0.0425) between VAP and no VAP.

^significant difference at D3 (p=0.0014) and D7 (p<0.0001) between VAP and no VAP.

Item S3. Univariate analysis - risk factors for VAP development.

|  | OR | lower | upper | 95% CI | p value |
| --- | --- | --- | --- | --- | --- |
| Age >60, years | 0.97 | 0.74 | 1.26 | 0.97 (0.74-1.26) | 0.557 |
| Gender, male | 1.39 | 1.10 | 1.76 | 1.39 (1.1-1.76) | 0.006 |
| BMI >30, kg/m^2^ | 1.08 | 0.80 | 1.44 | 1.08 (0.8-1.44) | 0.624 |
| History pulmonary disease | 1.49 | 0.80 | 2.79 | 1.49 (0.8-2.79) | 0.200 |
| History heart failure | 0.87 | 0.44 | 1.68 | 0.87 (0.44-1.68) | 0.678 |
| History arterial hypertension | 0.91 | 0.72 | 1.16 | 0.91 (0.72-1.16) | 0.457 |
| Active smoking | 1.40 | 1.07 | 1.82 | 1.4 (1.07-1.82) | 0.013 |
| Diabetes mellitus | 1.04 | 0.74 | 1.46 | 1.04 (0.74-1.46) | 0.823 |
| History of malignancy | 0.96 | 0.56 | 1.61 | 0.96 (0.56-1.61) | 0.861 |
| TBI | 1.10 | 0.88 | 1.37 | 1.1 (0.88-1.37) | 0.398 |
| ICH | 0.89 | 0.70 | 1.12 | 0.89 (0.7-1.12) | 0.314 |
| SAH | 1.33 | 1.00 | 1.76 | 1.33 (1-1.76) | 0.050 |
| IS | 0.66 | 0.44 | 0.99 | 0.66 (0.44-0.99) | 0.043 |
| CNS infection | 0.34 | 0.18 | 0.62 | 0.34 (0.18-0.62) | 0.000 |
| Brain tumor | 0.58 | 0.32 | 1.00 | 0.58 (0.32-1) | 0.051 |
| Lowest GCS | 0.82 | 0.65 | 1.02 | 0.82 (0.65-1.02) | 0.077 |
| Lowest GCS motor | 1.08 | 0.00 | 0.00 | 1.08 (0.00-0.00) | 0.017 |
| Anisocoria | 1.16 | 0.91 | 1.49 | 1.16 (0.91-1.49) | 0.229 |
| Intracranial probe | 2.33 | 1.86 | 2.92 | 2.33 (1.86-2.92) | 0.000 |
| Ventricular drainage | 1.57 | 1.24 | 2.00 | 1.57 (1.24-2) | 0.000 |
| Posterior fossa injury | 1.26 | 0.79 | 1.99 | 1.26 (0.79-1.99) | 0.322 |
| Therapeutic hypothermia | 4.24 | 2.39 | 7.97 | 4.24 (2.39-7.97) | 0.000 |
| Barbiturate coma | 1.29 | 0.81 | 2.04 | 1.29 (0.81-2.04) | 0.276 |
| Neurosurgery | 1.07 | 0.86 | 1.34 | 1.07 (0.86-1.34) | 0.535 |
| Decompressive craniectomy | 1.24 | 0.94 | 1.64 | 1.24 (0.94-1.64) | 0.129 |
| Country | 1.14 | 0.98 | 1.34 | 1.14 (0.98-1.34) | 0.000 |
| Tracheobronchitis | 2.27 | 1.57 | 3.33 | 2.27 (1.57-3.33) | 0.000 |
| Corticosteroids | 0.92 | 0.68 | 1.24 | 0.92 (0.68-1.24) | 0.600 |

BMI=body mass index, TBI=traumatic brain injury, SAH=subarachnoid hemorrhage, IS=ischemic stroke, CNS=central nervous system, ICH=intracranial hemorrhage, GCS=Glasgow coma scale.

Item S4. Logistic Cox regression model – univariate for ICU mortality

| UNIVARIATE | | | | |
| --- | --- | --- | --- | --- |
|  | beta | HR (95% CI for HR) | wald.test | p.value |
| VAP | -0.52 | 0.6(0.37-0.96) | 4.4 | 0.035 |
| AGE | 0.039 | 1(1-1.1) | 21 | 0.000 |
| GCSlowest | -0.002 | 1(0.91-1.1) | 0 | 0.970 |
| Anisocoria | -0.13 | 0.88(0.52-1.5) | 0.25 | 0.610 |
| Neurosurgery | 0.14 | 1.2(0.72-1.8) | 0.35 | 0.550 |
| ICP | -1.5 | 0.23(0.13-0.41) | 26 | 0.000 |
| EVD | -0.22 | 0.8(0.5-1.3) | 0.79 | 0.370 |
| Pulmonary | -1.1 | 0.33(0.046-2.4) | 1.2 | 0.270 |
| Cardiovascular | 1.5 | 4.3(2-9.5) | 13 | 0.000 |

GCS=Glasgow coma scale. CI=confidence interval, HR=hazard ratio, ICP=intracranial probe, EVD= external ventricular drainage, VAP=ventilator-associated pneumonia.

Item S5. Logistic Cox regression model – multivariate for ICU mortality

| MULTIVARIATE | | | | | | | | | | |
| --- | --- | --- | --- | --- | --- | --- | --- | --- | --- | --- |
|  | coef | HR=exp(coef) | se(coef) | z | Pr(>\|z\|) | exp(coef) | exp(-coef) | lower .95 | upper .95 | HR (95% CI for HR) |
| VAP | -0.34847 | 0.71 | 0.25 | -1.38 | 0.168 | 0.71 | 1.42 | 0.43 | 1.16 | 0.71 (0.43-1.16) |
| AGE | 0.029711 | 1.03 | 0.01 | 3.49 | 0.000 | 1.03 | 0.97 | 1.01 | 1.05 | 1.03 (1.01-1.05) |
| ICP | -1.17892 | 0.31 | 0.29 | -4.03 | 0.000 | 0.31 | 3.25 | 0.17 | 0.55 | 0.31 (0.17-0.55) |
| Cardiovascular | 1.012557 | 2.75 | 0.41 | 2.48 | 0.013 | 2.75 | 0.36 | 1.23 | 6.14 | 2.75 (1.23-6.14) |

CI=confidence interval, HR=hazard ratio, ICP=intracranial probe, EVD= external ventricular drainage. VAP=ventilator-associated pneumonia.

Item S6. Multivariable model - risk factors for ICU length of stay.

| Coefficients: | Value Std. | Error | t value | p value |
| --- | --- | --- | --- | --- |
| Male gender | -0.00885 | 0.11644 | -0.07597 | 1.061 |
| VAP | 0.91654 | 0.12809 | 7.15527 | 0.000 |
| Therapeutic hypothermia | -0.05572 | 0.26795 | -0.20794 | 1.165 |
| Lowest GCS motor score | -0.08299 | 0.13673 | -0.60698 | 1.456 |
| Lowest GCS at inclusion | -0.17965 | 0.13403 | -1.3403 | 1.820 |
| ARDS | 0.097638 | 0.19689 | 0.4959 | 0.620 |
| Invasive mechanical ventilation days | 2.093419 | 0.09328 | 22.44272 | 0.000 |
| Intercepts: |  |  |  |  |
| 0\|1 | -8.8534 | 8.9716 | -0.9868 | 1.676 |
| 1\|2 | 1.825 | 0.2195 | 8.3136 | 0.000 |
| 2\|3 | 4.1082 | 0.2479 | 16.5688 | 0.000 |
| 3\|4 | 7.2229 | 0.3164 | 22.83 | 0.000 |
| 4\|5 | 10.458 | 0.4149 | 25.2043 | 2.000 |

| Coefficients: | Value Std. | Error | t value | p value | OR | IC95% |  |  |  |
| --- | --- | --- | --- | --- | --- | --- | --- | --- | --- |
| VAP | 0.9361 | 0.12175 | 7.689 | 0.000 | 2.55 | [2.01-3.23] |  |  |  |
| Invasive mechanical ventilation days | 2.0804 | 0.08965 | 23.207 | 0.000 | 8.1 | [6.72-9.75] |  |  |  |
|  |  |  |  |  |  |  |  |  |  |
| Intercepts: |  |  |  |  |  |  |  |  |  |
|  | Value Std. | Error | t value | p value | OR |  |  |  |  |
| 0\|1 | -7.9104 | 6.251 | -1.2655 | 1.794 | 0.0004 |  |  |  |  |
| 1\|2 | 1.9844 | 0.1418 | 13.9923 | 0.000 | 7.2745 |  |  |  |  |
| 2\|3 | 4.2621 | 0.1812 | 23.5214 | 0.000 | 70.9621 |  |  |  |  |
| 3\|4 | 7.3556 | 0.2663 | 27.6258 | 0.000 | 1564.9850 |  |  |  |  |
| 4\|5 | 10.6173 | 0.3787 | 28.0378 | 0.000 | 40835.1500 |  |  |  |  |

VAP=ventilator associated pneumonia. ARDS=Acute respiratory distress syndrome. GCS=Glasgow coma scale.
